# Mammalian cumulus-oocyte complex communication: a dialog through long and short distance messaging

**DOI:** 10.1007/s10815-022-02438-8

**Published:** 2022-05-02

**Authors:** Mathilde Marchais, Isabelle Gilbert, Alexandre Bastien, Angus Macaulay, Claude Robert

**Affiliations:** Département des sciences animales, Centre de recherche en Reproduction, Développement et Santé Intergénérationnelle (CRDSI), Réseau Québécois en Reproduction (RQR), Pavillon Paul Comtois, Université Laval, Québec, QC Canada

**Keywords:** Folliculogenesis, Signal transduction, Cellular communication, Cumulus-oocyte complex, Transzonal projections

## Abstract

Communications are crucial to ovarian follicle development and to ovulation, and while both folliculogenesis and oogenesis are distinct processes, they share highly interdependent signaling pathways. Signals from distant organs such as the brain must be processed and compartments within the follicle have to be synchronized. The hypothalamic–pituitary–gonadal (HPG) axis relies on long-distance signalling analogous to wireless communication by which data is disseminated in the environment and cells equipped with the appropriate receptors receive and interpret the messages. In contrast, direct cell-to-cell transfer of molecules is a very targeted, short distance messaging system. Numerous signalling pathways have been identified and proven to be essential for the production of a developmentally competent egg. The development of the cumulus-oocyte complex relies largely on short distance communications or direct transfer type via extensions of corona radiata cells through the *zona pellucida*. The type of information transmitted through these transzonal projections is still largely uncharacterized. This review provides an overview of current understanding of the mechanisms by which the gamete receives and transmits information within the follicle. Moreover, it highlights the fact that in addition to the well-known systemic long-distance based communications from the HPG axis, these mechanisms acting more locally should also be considered as important targets for controlling/optimizing oocyte quality.

## Introduction

In the ovarian follicular compartment, different cell types interact with each other in ways that are so interdependent that the follicle has been compared to a syncytium [1, 2]. The finality of folliculogenesis from recruitment to ovulation is to support the production and liberation of a developmentally competent egg [3]. This is ensured by making the gamete the focus of follicular function. The presence of the oocyte is essential for follicular survival and synchrony of the different follicular compartment is ensured by intercellular communications [4–6]. This perfect synchronism still represents a key challenge to control and track via the monitoring and modulating of the systemic/long-distance communications. Following ovulation, most of the cells left behind in the follicle will form the *corpus luteum* and will continue to remotely support the developmental fate of what was once the oocyte by initiating a dialog with the tissues surrounding the conceptus [7]. Coordinating this entire process requires an intricate network of intercellular communication [8, 9].

Structurally, the ovarian follicle is highly segmented with definite compartments [9]. A more diffuse structure could ease communications between cell types but different steps along folliculogenesis are inducing a physical separation between cellular lineages [10]. At the antral stage where all cell types are present, theca cells are the most external relative to the gamete [11]. As they are separated from the follicle by the basal lamina, they do not physically interact with the cells inward the follicle. On the internal side of this basal lamina, different types of somatic cells are forming the contingent of granulosa cells [8]. Further distinctions are made between the granulosa cells lining the antrum, and those supporting the oocyte, called cumulus cells, as well as the layer surrounding the *zona pellucida*, sometimes called the *corona radiata* [12]. Although all granulosa cells originate from the proliferation of the pre-granulosa cells surrounding the oocyte in the primordial follicle, they show distinctive morphology [12]. Mural granulosa cells are found in a thin layer whereas cumulus cells form a thicker cloud (hence the name) over the oocyte while *corona radiata* cells are in close proximity to the oocyte and display cellular extensions across the *zona pellucida* reaching and contacting the *oolemma* [13]. Aside from the contact from these transzonal projections, the oocyte itself is isolated within the shell of glycoproteins [14].

Given that all compartments are essential to successful folliculogenesis and oogenesis and that to be successful they must remain in synch along all their specific proliferation, growth, and differentiation phases, inter-compartment communications must be efficient, well-regulated, and multi-directional [8]. These considerations are underlining the complexity of intra-ovarian communications, but the female reproductive cycle is a complex process also remotely controlled by the hypothalamic–pituitary–gonad axis [9]. This long-distance communication between the brain, other organs, and the ovary is based on secreted factors transported through the blood stream and disseminated across the surrounding environment to be interpreted by the targeted receiver. Numerous growth factors, metabolites, and ions that act upon the ovarian follicle using a signal transduction going from a reception in the outer follicular compartments and transmission inward towards the oocyte [8, 15]. This external long-distance signaling is then interpreted and re-emitted differently to maintain and modulate delicate physiological functions, each of which appear to determine some aspect of oocyte quality. In mono-ovulatory species, most follicles will never reach ovulation and the gamete inside will simply die [16–18]. Based on the foregoing description, the gamete is the endpoint of the follicle and could be considered as a passive passenger totally dependent on the nurturing environment provided by the follicular cells. However, it has been shown that the oocyte plays a critical and active role in supporting folliculogenesis and that its premature demise or disruption of its signals leads to follicular atresia [8, 19]. For example, oocyte-secreted factors such as GDF9 and BMP15 are known to play essential roles in the growth and differentiation of granulosa and cumulus cells [20–22]. Fushii et al. have demonstrated that mural granulosa cells cultured in presence of denuded oocytes are somehow drawn to establish direct transfer communication through the establishment of TZP in a manner similar to that of cumulus cells [23]. This well illustrates the extent of the interdependence between the compartments within ovarian follicles, as well as the requirement for two modes of intercellular communication observed so far: secreted and direct transfer.

This long-distance, systemic signaling and control of follicular development is instrumental for modulating fertility and carrying out clinical interventions. However, the well-being of the follicle depends on the intrafollicular interpretation and re-emission of these external signals across the follicular compartments, which rather rely on direct transfer signaling [8, 15, 24]. This complex intrafollicular dialog occurring through the diffusion and exchange of small labile compounds such as steroid and peptide hormones, metabolites, and ions has been described but has not yet been fully elucidated [8, 15]. At the hearth of the follicle, the oocyte relies on the surrounding cumulus cells to communicate through direct transfer of small and large molecules [5, 6, 13]. Recent findings indicate that ribonucleoprotein complexes are delivered as the contents of extracellular vesicles, or through direct contact between neighbouring cells [25–28]. In the latter case, even larger parcels such as organelles might be transferred. The modes of intercellular communication observed so far are divided into two categories: secreted and direct transfer. The present review provides a brief and broad overview of the communication routes that are known to influence ovarian functions and then focuses on the direct cell-to-cell communications occurring within cumulus-oocyte complexes. This review aims to initiate a reflection on the importance of these intimate direct communications between the innermost cell types that are the *corona radiata* and the oocyte which may be representative of the final interpretation of the global messages. These direct communications may be considered as potential targets to modulate oocyte’s development and quality. Clinical interventions mainly act on the HPG axis, but the overlay of the different types of messaging and incoming messages reaching the follicle are all different endogenous means by which the final interpretation may deviate in an unexpected/undesired manner.

### Long-distance secreted communications from the outside

#### Endocrine signaling

The systemic dialogue between the female reproductive organs has been studied extensively [29]. Most of the focus has been given to the antral phase of folliculogenesis where all somatic cell types are present and when the follicular unit is highly responsive upon the control operating remotely through the hypothalamic–pituitary–gonadal axis (HPG axis). Since signalling occurs via the bloodstream, it was already possible several decades ago to monitor fluctuations of hormones and correlate these with physiological responses. Much has been learned from the variability that exists within and across species to the point where we can finely tune the estrus cycle to optimize or prevent ovulation in human health or in commercial animal reproduction. Current understanding of the general concept is provided here to characterize the framework in which the cumulus-oocyte complex develops.

Early folliculogenesis is often thought to be under an autonomous developmental program as follicular recruitment and growth up to the stage of secondary follicle can be sustained in absence of gonadotropins [30]. However, in vitro culture of ovarian follicles indicates that low dose of FSH is beneficial [31]. Therefore, the pre-antral stage may not require FSH but its presence seems beneficial. New studies continue to emerge revealing the importance of the complex endocrine signaling that governs the various processes of folliculogenesis.

In endocrine signalling, a hormone (steroid or protein) secreted into the bloodstream is transported through the entire body often attached to a carrier such as albumin in search of its specific receptors on the surface of targeted cells, which then respond in specific ways. The hormone molecule is thus a vehicle of information transfer or an instruction. It has been shown that many organs and glands far from the ovary maintain a bidirectional dialogue through this whole-body messaging system. The best known is the brain-gonad dialogue, which exemplifies the two-cell-two-hormone (gonadotropin) theory [32].

The action starts in the brain, with the hypothalamus secreting gonadotropin–releasing hormone (GnRH). Through the hypophyseal portal system, GnRH reaches the anterior pituitary gland, where it regulates the release of gonadotropins such as follicle-stimulating hormone (FSH) and luteinizing hormone (LH), which are secreted into the bloodstream. By binding to ovarian receptors, LH stimulates theca cells to produce androgens, and FSH stimulates granulosa cells to convert androgens into oestrogens through the action of aromatase [32]. The oestrogens thus produced enter the bloodstream and reach the brain, where they exert negative feedback control over gonadotropin secretion. The ovaries are therefore also endocrine glands. At a certain point, oestrogen levels reach a threshold that triggers an LH surge, constituting a positive feedback signal that induces ovulation and stimulates the development of the *corpus luteum* and of the uterine endometrium [7]. This textbook model is illustrative of how ovarian follicles are influenced strongly by external cues. These messages take the form of protein hormones or steroids which are more labile as they can more easily cross cellular membranes. The duration of the signal transmission depends upon many factors including the molecule’s half-life and bioavailability which are in turn affected by the stability of the molecule and the presence of carriers in addition to the metabolic clearance rate, which is influenced by the activity of catabolizing enzymes, sequestering potential by adipose tissue in the case of steroids, etc. For instance, there are different FSH isoforms whose half-life is variable (between three to four hours) and dependent on the composition of two carbohydrate groups, attached to each subunit [33]. Although LH and FSH share same alpha subunits, the two hormones differ in the composition of their carbohydrate moieties attaches to their beta subunits, explaining the shorter half-life of LH (20 min) [33]. It is known that follicles receive information not only from the brain but also from other tissues such as the thyroid gland [34], the adrenal glands [35], and even the adipose tissue [36, 37]. All these signals are then channelled to various compartments, including the cumulus-oocyte complex.

### Intrafollicular secreted signal transmission

#### Autocrine and paracrine signalling

Once a hormonal message reaches the ovary, it is conveyed towards the gamete and in some cases amplified through autocrine and paracrine signalling within the follicle. The dual requirement of the presence of both FSH and soluble secreted factors for the expansion of mouse cumulus oocytes complexes is a good example of crosstalk between signaling pathways [38]. Much local cell-to-cell dialogue has been identified, often involving members of the transforming growth factor beta (TGF $\beta$) family (inhibin, activin, anti-Müllerian hormone, growth and differentiation factor 9) and bone morphogenetic proteins [39]. All such dialogues have been shown to be crucial for proper follicular development from follicle assembly to ovulation.

Communication routes and the information communicated thereby, often change during folliculogenesis. Intercellular communication during pre-antral growth appears to be more rudimentary than at later stages. Cell responsiveness increases concomitantly with the formation of the antrum, during which granulosa cells differentiate into mural cells (distal from the oocyte) and cumulus cells (proximal to the oocyte). As the follicle diameter (and hence the distance between the farthest granulosa cells and the gamete) increases, so does the importance of paracrine signalling from outer cells towards the cumulus-oocyte complex via the follicular fluid. Prime examples of this are the closely related protein dimers that make up inhibin and activin, which mediate intra-ovarian communications as well as systemic effects [40, 41]. Another example of paracrine signalling is the release of EGF-like peptides by granulosa cells (by proteolytic cleavage of preproproteins) into the follicular fluid for specific receptors expressed on cumulus cells [42]. Moreover, the implication of neurotrophins (NTs) (nerve growth factor (NGF) or brain-derived neurotrophic factor (BDNF)) in the assembly of growing ovarian follicles, their growth, and their survival is also an instance of this important intercellular communication [43–45]. These paracrine or wireless signalling pathways via follicular fluid convey essential information to the innermost cells and supports an intense metabolic activity [46]. How these communication routes are integrated is not yet fully understood. Some appear to be redundant or to allow crosstalk between stimulatory signals. These aspects may turn out to be mechanisms of signal amplification.

### Vesicles and exosomes

In addition to paracrine secretion of proteins or peptides into the follicular fluid during antral folliculogenesis, growing evidence indicates that granulosa cells also secrete membrane-enclosed bodies containing substances that influence the function of recipient cells. The production and release of extracellular vesicles are widespread among somatic cells. Three main types of vesicle have been defined [47–49]: (1) exosomes (30–150 nm), (2) apoptotic bodies (800–5000 nm), and (3) ectosomes/microparticles/microvesicles (100–1000 nm). Extracellular vesicles are thus highly heterogeneous in size, but also in membrane composition, biogenesis, and surface markers [47], making their classification complex. Their location (extracellular or intracellular), surface protein markers (e.g., tetraspanins, coat proteins I and II, COPI and COPII), and clathrin-dependent or clathrin-independent status are the usual criteria [50]. Despite their morphological and biological differences, both microvesicles and exosomes are able to take on and transfer different macromolecules from and to recipient cells [51, 52].

Among the molecules found in extracellular vesicles, microRNA is the best described. By their strong stability and their resistance to degradation, microRNA play an important role in effects of extracellular vesicles on cells (reviewed by [53]). MicroRNA-containing vesicles have been found in follicular fluids in a wide variety of species [25, 51, 54–57]. Navakanitworakul and his collaborators have shown that these extracellular vesicles change in number and in their small RNA content across folliculogenesis [25]. Extracellular microRNA present in follicular fluid has been implicated in numerous intercellular communications between ovarian cells [58]. Some forms delivered by extracellular vesicles appear to be involved in promoting gamete maturation [50]. In fact, Santonocito et al. [51] have found that miR-99a, miR-100, miR-132, and miR-218 could be involved in follicle maturation while miR132, miR212, and miR214 could be able to trigger meiosis resumption by negatively regulating genes encoded for follicle maturation-inhibiting factors and miR29a could be involved in epigenetic modifications (reviewed by [59]). A recent study showed that canine microvesicles contain miR-30b, miR 375, and miR 503 [60]. These miRNAs are involved in various pathways such as WNT, MAPK, ERb $\beta$, and transforming growth factor beta (TGF $\beta$) which are closely related to follicular growth and oocyte maturation [51, 55, 57, 61]. Moreover, it has been demonstrated that extracellular vesicles content in aging mares can negatively impacts TGF $\beta$ family members, resulting in compromised oocyte maturation [62]. Furthermore, granulosa and cumulus cells have been shown to take up extracellular vesicles and cumulus cell expansion is promoted in their presence [26, 27]. The presence of extracellular vesicles in follicular fluid is also beneficial to cumulus-oocyte complexes by protecting it from the harmful effects of heat shock stress [63]. Following its upregulation by oocyte-secreted factors including GDF9, miR21 rescued granulosa cells from undergoing apoptosis [64, 65]. By its higher proportion in cumulus cells of oocytes that developed into blastocysts, miR-21 is linked to oocyte developmental potential and therefore blastocyst formation [66]. Another way to measure oocyte capability to develop to the blastocyst stage is linked to lipid content bundled in extracellular vesicles, found in follicular fluid [67]. At this point, it is still unclear if such stimulatory effects are triggered by internal contents or by surface-bound molecules of extracellular vesicles found in follicular fluid.

Despite all of these discoveries, to date, our knowledge about intercellular communication mediated by exosome or microvesicles inside the ovarian follicle is limited and many questions remain [59]. Among these questions, it remains to determine which macromolecules, in addition to miRNAs, are carried out by extracellular vesicles. It has been shown in other cell types, namely platelets, that microvesicles can be formed from lipid raft domains present on the plasma membrane of communicating cells [68]. Rafts are mainly active and selective association of cholesterol, sphingolipids proteins, and lipids [69]. They are involved in cell adhesion, membrane trafficking, and signal transduction events [69]. In reproductive physiology, lipid rafts have been studied in sperm cells [70] and in relation to the fusion of spermatozoon and oocyte membranes [71].

To date, intercellular communication mediated by microvesicles, exosomes, microRNA, and lipid rafts remain largely unexplored in the follicle. Many questions about their regulation remain unanswered and identification of complete exosome cargo could provide information on key regulators involved in signaling pathways relevant to oocyte, embryo, and fetus healthy development.

### The ovarian secretory transmission

The communication between the oocyte and its nurturing cells is essential for the nuclear regulation of the oocyte and its subsequent development capacity [72]. Once considered a passive element, the oocyte is now known as the driving force of this relationship [15]. With the secretion of soluble factors, the oocyte will therefore actively regulate the functions of the granulosa cells and the cumulus cells related to growth and differentiation of somatic cells [73]. Important processes regulated by these oocyte-secreted factors (OSFs) include the regulation of granulosa and/or cumulus cell proliferation and follicular growth rate [74, 75], glycolysis promotion by cumulus cells, necessary for oocyte metabolism [76], the acquisition of signalling capabilities of EGF molecules by cumulus cells, required for ovulatory cascade recognition by the COC [77, 78], and control of mucification and expansion of cumulus cells necessary for ovulation [38, 79]. These OSFs appears to be the source of signals that are essential to the proper development of its follicle, and hence to its own competence [8, 75]. The most studied OSFs are growth differentiation factor 9 (GDF9), GDF9b (often referred to as bone morphogenetic factor 15 or BMP15), BMP6, and various fibroblast growth factors [8, 75]. In females, GDF9 and BMP15’s expression is largely restricted to the oocyte where they are co-expressed throughout most of folliculogenesis [8]. GDF9b in conjunction with GDF9 through autocrine and paracrine signalling have been shown to regulate the growth and differentiation of granulosa and theca cells, which in turn influence oocyte developmental competence [19, 80]. By its action on granulosa cells morphology, recent studies suggest that GDF9 might promote filopodia generation in outgrowing granulosa cells, which penetrated the oocyte and provided a foundation for oocyte-granulosa/cumulus cell communication [81, 82].

This bidirectional communication is well exemplified by the interplay between GDF9 and the kit ligand (KITL). GDF9 secreted by oocytes promotes both proliferation and differentiation of granulosa cells in vitro [22, 83, 84]. In a *gdf9* null mutant, oocytes develop atypically and eventually degenerate [21, 85]. Conversely, granulosa cells secrete KITL, which binds to the c-kit receptor expressed by theca cells and the gamete and stimulates the growth of the latter [86]. GDF9b/BMP15 secretion in turn reduces KITL expression, which leads to increased expression of the FSH receptor on granulosa cells, which then become more responsive to the signal to proliferate [87].

GDF9 signalling has been shown to regulate granulosa and thecal cell growth, differentiation, and function through autocrine and paracrine mechanisms. This communication maintains the delicate balance between growth and differentiation both in oogenesis and folliculogenesis, which fall into dysphasia when asynchrony arises between follicular compartments, resulting in the failure to yield a full-grown and developmentally competent ovule.

### Direct transfer communication

Whereas systemic long-distance secretory communication is based on dissemination of information to any receiver capable of picking up the signal via specific receptors, direct communication relies on direct transmission via physical contact. In an ovarian follicle, theca cells are physically isolated from the other cell types by the basal lamina, which prevents direct exchange of material with the inner structure. In contrast, sheets of granulosa cells are in direct contact with each other and eventually mingle with what become the cumulus cells surrounding the oocyte. Although myriad intracellular pathway signals are transduced in wireless mode, for example, WNT/β-catenin [88], SMADs [89], PI3K-AKT-PTEN [90], TSC-mTOR [91], and MAPK-ERK [64], transmission of physiological cues throughout the syncytium all the way to the oocyte by direct cell-to-cell transfer of material through intercellular bridges has not been confirmed. Such communication routes are difficult to observe because of the juxtaposition of the plasma membranes.

### Cumulus-oocyte interdependence based on physical contact

From the basal folliculogenesis, a dialogue is established between the oocyte and the somatic cells of the follicle [15]. Material transfers between cumulus cells and the gamete are a known requirement for oocyte growth and maturation [15, 75]. The extent of the cumulus-oocyte interdependency was shown initially as functional impairments observed in the cumulus cells when the oocyte was removed from the complex [92]. This communication was then shown to be in the nature of direct transfer at least in part, since stripping the somatic cells from the gamete significantly impacted the oocyte’s developmental competence even though all the cells were still in the dish [93, 94]. It is now well established that cumulus cells must be in direct contact with the gamete in order to bring about the resumption of meiosis and provide metabolic support. Among the most often cited transferred molecules are the cyclic nucleotides cAMP and cGMP [95, 96], amino acids [97–99], and energy substrates, primarily lactate, pyruvate, and phosphocreatine [100–103].

At its full size, the oocyte is the largest cell in the body, and its cytoplasm has an atypical composition that includes mRNA and protein reserves and a large number of immature mitochondria that individually have limited energy-generating capacity [104–106]. Their capacity for glucose uptake is also limited [100, 101], making the gamete dependent on support from cumulus cells. In return, the oocyte contributes to cumulus cell function via paracrine signalling by secreting OSFs such as GDF9 and GDF9b/BMP15 [75]. Oocyte-derived GDF9b/BMP15 and FGFs cooperate to promote glycolysis in cumulus cells [76] and thereby provide energy substrates to the oocyte. The purpose of delegating such an important role to surrounding cells remains unclear, but the mutual interdependency is a fundamental aspect of oocyte development and fertility.

### Transferring small loads: gap junctions

The communication between the oocyte and follicle somatic cells, indispensable both for oocyte’s growth and maturation and for the development of the follicle itself, is realized partly through the gap junctions [4]. It is now well established that the oolemma and the plasma membranes of *corona radiata* cells are intact at their connexion points. So the direct involvement of the membranes on both sides of the zona pellucida implies limits on potential exchanges of material. Trans-membrane proteins called connexins, which can be assembled to form connexons or hemi-channels, are abundant at both ends of the membranes [107, 108]. Once aligned, connexons facing each other form gap junctions. These tiny intercellular channels are well known in cumulus-oocyte complex [109]. Their pore size allows direct transfer of substances of mass smaller than 1 kDa; hence, ions, metabolites such as pyruvate, lactate, phosphocreatine, and amino acids, secondary messengers such as cAMP, cGMP [110, 111], and even electrochemical potentials [112]. The latter play an important role in the regulation of meiosis [72] and are therefore affected by ovulation-related events.

Many connexins (e.g., Cx26, Cx30, Cx32, Cx37, Cx40, Cx43, and Cx45) have been detected in ovarian cells, and Cx37 and Cx43 in particular have a major influence on folliculogenesis and play a crucial role in fertility [109, 113]. Connexons of precise pore size can be made of different connexins, as seen in mice, or the same type, as often observed in cattle [114, 115]. Moreover, in the cumulus-oocyte complex, gap junctions are essential components and control the resumption of meiosis through management of oocyte cAMP and cGMP concentrations. These cyclic nucleotides are transferred from cumulus cells to the oocyte and maintain meiotic arrest at the prophase I stage until the oocyte is ready to undertake the ovulation process [116–118]. Although species specificity has been reported, the general model considers the control by cumulus cells over meiosis as part of downstream signalling by gonadotropins. Whereas FSH promotes communication through gap junctions, the LH surge has an opposite effect, generally causing disjunction and halting cAMP transfer as well as activating phosphodiesterase 3 to deplete intracellular cAMP [117, 119]. In vitro experiments have shown that in pig oocytes, the protein regulated by the LH surge is connexin cx43, while other connexins remain unaffected [120]. In mice, LH causes gap junction closure [121], whereas in cattle a disjunction occurs, which initiates post germinal vesicle breakdown [122].

Cumulus cells also transfer energy substrates such as lactate, pyruvate, NADPH, and amino acids and purine substrates such as phosphoribosyl pyrophosphate (PRPP) via gap junctions to satisfy the large metabolic demand of the oocyte [78].

Other molecules have also been reported to pass through gap junctions. Using morpholino probes, it has been shown in cell culture that small interfering RNA (siRNA) passes through junctions made of Cx43 (but not Cx32/Cx36) for successful knockdown of the targeted gene [123, 124]. In these experiments, shorter sequences were faster and more effective, and sequences longer than 24 bp were less likely to pass, perhaps because of their secondary structure. The transfer even of small RNA was unexpected.

### Intercellular direct transfers: transzonal projections

Gap junctions ensure contact between the follicular somatic cells but also between cumulus cells and the oocyte, by being present on the TZPs, which are long cytoplasmic extensions of cumulus cells that pass through the *zona pellucida* and affix to the plasma membrane of the oocyte [24]. During the early stages of folliculogenesis and oogenesis, the primary follicle develops the *zona pellucida*, a shell-like layer of glycoproteins that forms a boundary between the gamete and the surrounding cumulus cells. The layer of cells in direct contact with the outer surface of the *zona pellucida* then develops transzonal cytoplasmic projections or TZPs, filipodia-like surface extensions that maintain communication with the gamete[125–127]. This layer constitutes the *corona radiata*. TZPs contain actin and/or tubulin backbone and a cytoskeleton [24]. However, their precise makeup and the extent to which they may be specialized remain unknown. They form relatively wide channels up to 2 µm in diameter [5] with bulging ends anchored to the oolema surface through peripheral microvilli that bear adherens-like junctions [5, 127, 128]. Moreover, catenin and cadherin proteins interacting with the actin cytoskeleton appear to be involved in anchoring the structures [128]. Although the precise assembly and maintenance mechanisms of junctions and TZP with oocyte membrane are unknown [129], new results suggest that embryonic poly(A)-binding protein (EPAB) [130] and focal adhesion kinase 2 (PTK2) [131] are important for bidirectional communication in COC, by establishing or maintaining TZPs and gap junctions at the pre-antral stage of folliculogenesis.

The establishment of this dense network of TZPs provides an interconnection within the *corona radiata*. In some species, this network is relatively straight in some and convoluted in others. Serial scanning electron microscopy images of ultra-microtome sections have shown that in mice, some TZPs are branched and not all of them reach the oocyte [127]. In cattle, the TZP network appears straighter overall, and the vast majority of projections reach across the zona pellucida and are in contact with the oolema [5].

The dynamics of TZP formation and establishment of the network is not yet well understood. It has been reported that projections increase in number as the oocyte grows [24] and GDF9 signalling from the oocyte seems to be involved [24, 82, 132]. El-Hayek et al. [82] suggest that the increased mRNAs encoding key structural components of filopodia by GDF9 promote the generation of new TZPs, enabling more efficient transfer of metabolites from the granulosa cells to the oocyte. However, other molecules may also govern TZPs’ formation, independently or co-operatively with GDF9 [82]. For instance, in *FSHb* null mice, stimulation with FSH increases the number of actin-containing TZPs, cell interconnectivity, and oocyte developmental competence [132], but the effect of FSH on tubulin TZPs is different. In these mice and in prepubertal mice, it causes retraction of these TZPs from the zona pellucida [133]. Active retraction is part of the process following meiosis resumption where TZP disconnection and the reduction of oocyte volume will create the perivitelline space between the *zona pellucida* and the oocyte oolemma [5, 134, 135]. While EGF was shown to increase the rate of TZP withdrawal [136], addition of 17 $\beta$ estradiol in bovine oocytes culture media [134] and FSH in porcine oocytes media [135] promotes TZP maintenance and acquisition of meiotic competence. Acting more upstream, it has been shown that resveratrol plays a critical role in regulating TZP assembly by promoting calcium ion transport into the cytosol to activate CaMKII $\beta$ phosphorylation essential to TZP synthesis [137]. Excessive CaMKII $\beta$, as seen in PCOS patients, results in failed TZP synthesis and damaged bidirectional communication between oocytes and granulosa cells [137].

### Transferring large loads: synaptic vesicular secretion

**Fig. 1:**
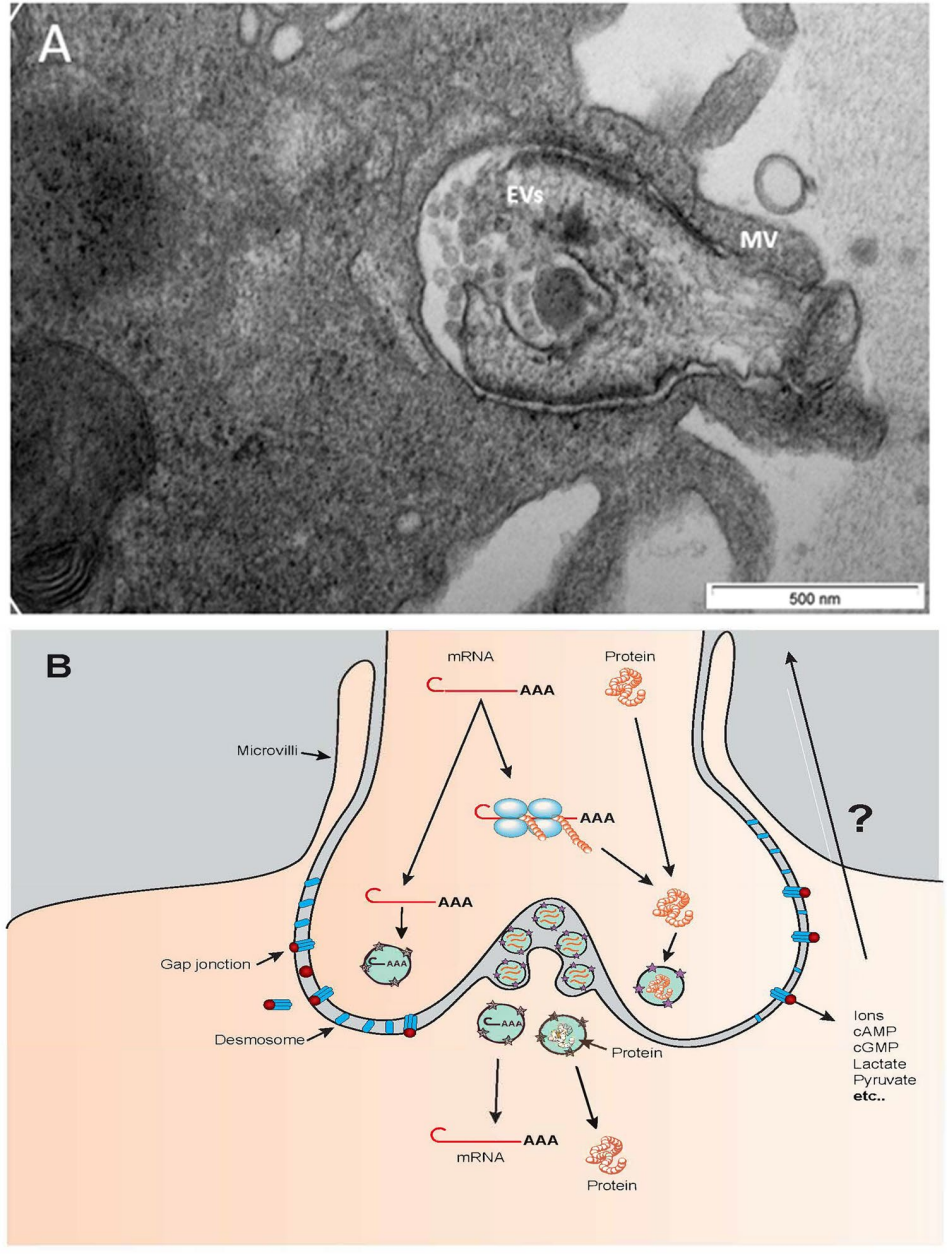
**A** Electron microscopy image of a bovine TZP terminus, the bulged portion held in place by microvilli (MV). Numerous extracellular vesicles (EVs, spheres about 50 nm in diameter) are visible. The projection terminus contains electron-dense structures. **B** Schematic representation of material movement through a TZP terminus. Transcripts (mRNA) could be transferred to the oocyte or translated polyribosomes, and proteins could be transferred. Small molecules could be transferred through gap junctions

In fact, from the primordial stage, the follicular cells surrounding the oocyte not only produce growth factors and hormones but provide the oocyte with physical support, nutrients, metabolic precursors, and other small molecules ($<$ 1 kDa), which can balance between compartments without affecting the distinctive macromolecular phenotype of either cell [138, 139]. Until recently, it was not believed that cumulus cells and oocytes exchanged substances of large molecular mass. It was then shown in maturing cattle oocytes that mRNA is shuttled via TZPs and accumulates [5]. The presence of poly-A tails and the co-localization of poly-A binding protein confirmed that these molecules were protein-encoding transcripts. This mRNA’s accumulation did not occur when the cumulus cells were stripped from the oocyte. In addition, transcription in the gamete nucleus was halted due to chromatin condensation. It was also shown that mRNA newly transcribed in cumulus cells were sent to TZPs and that these transcripts differed somewhat from cytoplasmic mRNAs, suggesting a selective passage of transcripts [5, 6]. The distribution of labelled RNA was polarized towards the plasma membrane. It was further observed that cumulus cells stripped from an oocyte reconnected via TZP remnants and that transcripts from GFP-transfected cumulus cells could be detected in the oocyte within hours of placing the cells together. Examination of TZP ultrastructure revealed vesicular secretion analogous to the cellular exchanges observed in the neuronal synapse [5, 6]. However, the mechanisms underlying the sorting, packaging, and transport of these large molecules remain to be elucidated. It also remains to be determined if mRNA is shuttled to the end of the cellular projections for onsite translation while proteins are packaged and exported to the neighbouring cell through vesicular secretion (Fig. 1), as is the case in neurons [140]. In bovine, it was shown that RNA accumulation within the TZPs is initiated in ovaries collected post-mortem even when the oocyte is still within its follicle and well under the mechanism presenting meiosis resumption [5, 6]. In fact, the timing of RNA accumulation within the TZPs in COCs still enclosed in the follicle fits with the timing of the acquisition of developmental competence [5, 6, 141]. This suggests that large cargo transfer may be part of the long sought-after process by which oocytes acquire developmental competence.

The transport of other large molecules through TZPs has since been shown in cattle oocytes. Cumulus cells are known to capture lipids from their microenvironment and fatty acid–binding protein 3 (FABP3) has been detected in TZPs, suggesting strongly fatty acid transport from cumulus cells to the oocyte during maturation [142]. Embryonic poly-A-binding protein (EPAB) has been found in TZPs in mice, and null mutants (*epab-*) are infertile, suggesting a crucial role of this protein in cumulus-oocyte complex function [130].

**Fig. 2:**
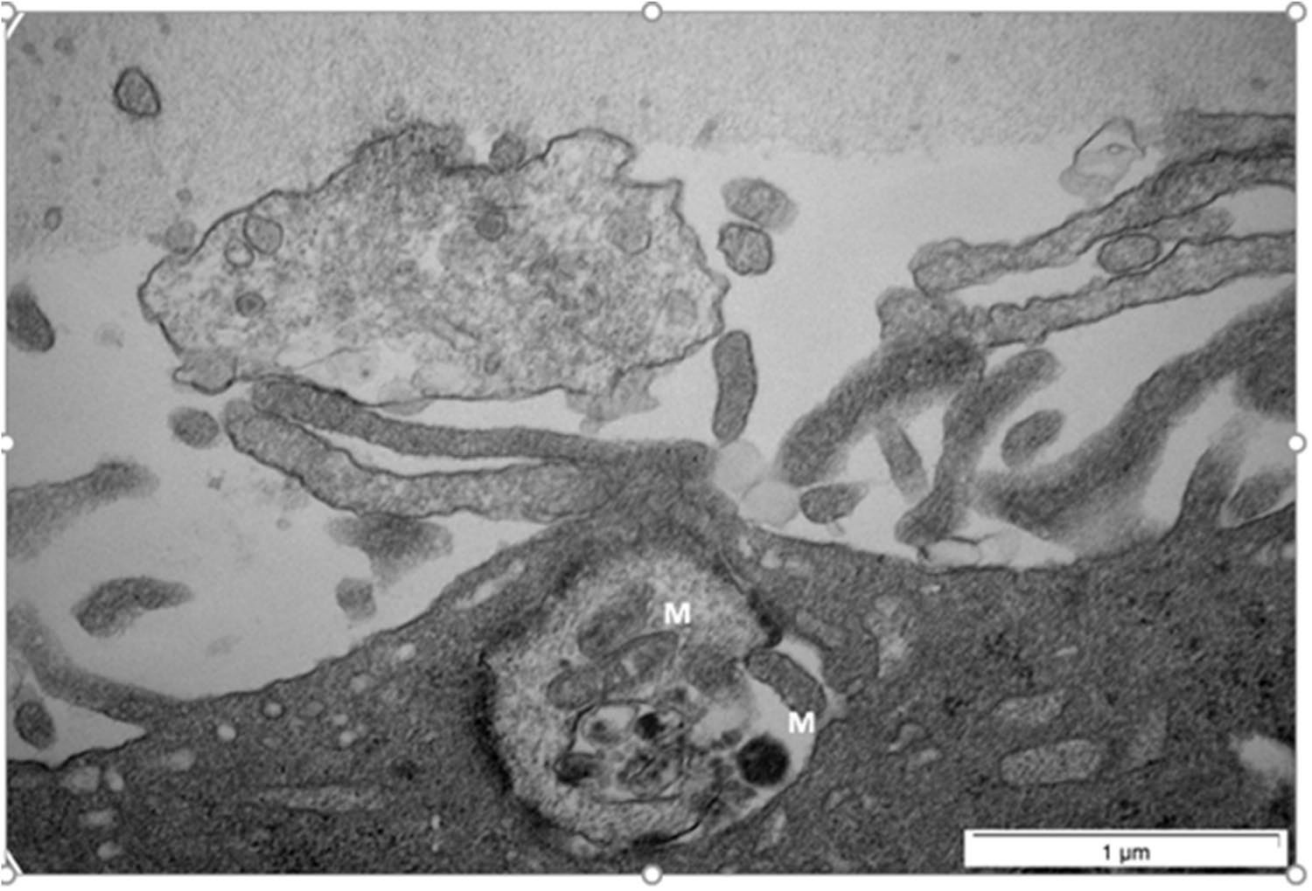
Electron microscopy image of a bovine TZP containing mitochondria (M)

**Fig. 3:**
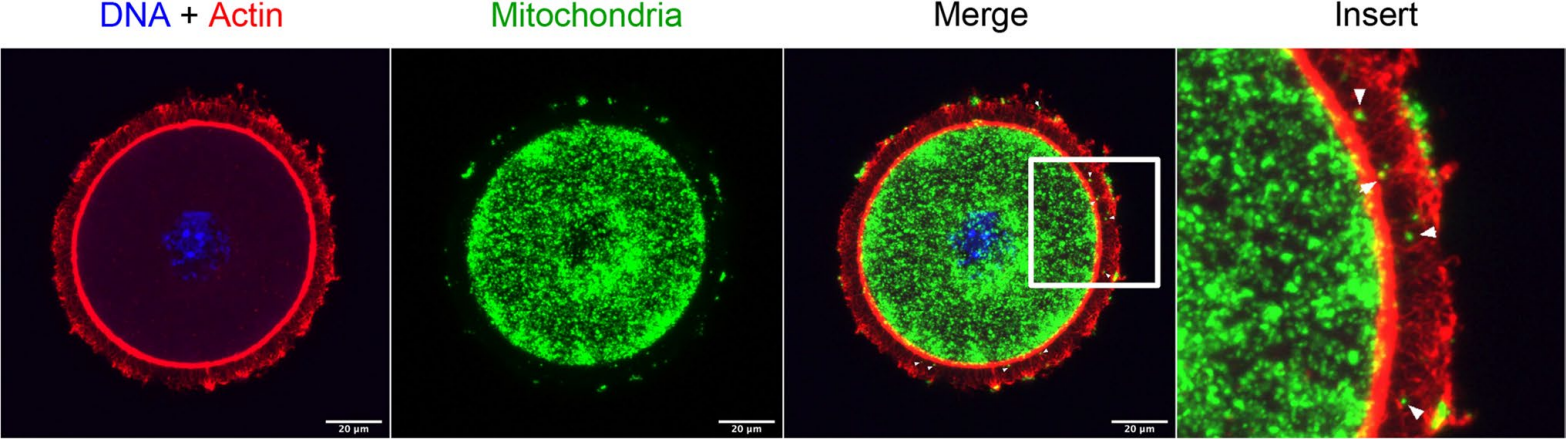
Mitochondria distribution in cumulus-oocyte complexes (COCs) in transgenic mice expressing mitochondrial-targeted red fluorescent protein. Immunofluorescence confocal images showed the presence of mitochondria throughout the cytoplasm with an accumulation at the cytoplasmic end of the oocyte, adjacent to the edge of the zona pellucida but also around the nucleus. Some points are found in the zona pellucida and at the periphery of cumulus cells. DNA material was stained with Hoechst 33,342 dye (blue) and actin with SiR-actin (red). Scale bar = 20 $\mu$ m

The transfer of large molecular masses within the cumulus-oocyte complex needs further study. The structure of the interface between the TZP and the oocyte have been compared to a neuronal synapse [5], with the bulging end constituted of numerous electron-dense structures, some of which are granular and others shaped like multi-vesicular bodies (Fig. 1A). Populations of small vesicles are often found at the interfaces of both cell membranes. The nature and roles played by these structures remain unknown, but they could be highly active domains that process the molecules shuttling between the two cells. Messenger RNA might be transferred to the oocyte or translated on ribosomes located at the tip of the TZPs, whereas proteins would be transferred directly to the oocyte (Fig. 1B). It is possible that other yet unidentified large masses could be downloaded to the oocyte. Transfer of even larger components such as organelles (e.g., mitochondria) cannot be ruled out (Figs. 2 and 3). No strong evidence of mitochondrial transfer between cumulus cells and the oocyte has come forth to date, but such exchanges have been reported in other cell types [143, 144]. We recently performed confocal imaging in cumulus-oocyte complexes of a line of transgenic mice expressing the DsRed2 fluorescent protein specifically targeting mitochondria. Representative confocal images show the presence of mitochondria throughout the cytoplasm of the oocyte with an accumulation at the cytoplasmic end of the oocyte, adjacent to the edge of the zona pellucida but also around the nucleus and some points are found in the zona pellucida and at the periphery of cumulus cells (Fig. 3).

Recently, it has been proposed that oocytes may expedite materials of considerable size to cumulus cells [145]. Molecules too large to pass through gap junctions have been shown to diffuse to granulosa cells in early-stage follicles and to cumulus cells in later stages after injection into the gamete cytoplasm. While the download of large molecules to the oocyte might be explained by the need to build reserves in the expectation of the demanding journey that is early embryogenesis, this uploading from the oocyte to the cumulus cells is intriguing and requires more investigation.

### Intercellular bridges among somatic cells

Exchanges of large loads between follicular cells might in fact be commonplace. This may have been overlooked because intercellular bridges are fragile. As mentioned above, not all corona radiata cell membrane projections penetrate the zona pellucida. Some remain outside the glycoprotein shell and are oriented towards other cumulus cells, suggesting additional direct communications throughout the ovarian follicle [127]. A similar situation occurs in sperm stem cells, which grow in clones that remain physically connected through short intercellular bridges that exhibit some plasticity and dynamism and apparently allow the exchange of large molecules [146].

### Tunnelling nanotubes

In other cell types, intercellular bridges called tunnelling nanotubes have been described. These are structures having a diameter of 50–200 nm, that is, at least 10 times smaller than TZPs [147, 148]. They allow exchanges of proteins, viruses and some organelles[149–151]. Their structure comprises actin filament backbone not unlike that of TZPs. However, they are open-ended and transient, existing for a few minutes up to several hours [147, 149], whereas TZPs are established days before their disconnection upon resumption of meiosis.

### Interactions between somatic cells and oocytes in non-mammalian species

Until recently, intercellular communications within mammalian cumulus-oocyte complexes did not appear to include transfers of macromolecules. However, numerous examples of somatic cells providing such support to gametes are well documented in other organisms. For example, folliculogenesis and oogenesis in *Drosophila* involves 15 nurse cells that are interconnected through cellular bridges [152]. As oogenesis reaches completion, the nurse cells empty their contents (maternal RNA, proteins, and organelles) into the oocyte, a process called nurse cell dumping. A mesh of actin filaments supports the transfer and retains the nuclei of the nurse cells [152]. In *Caenorhabditis elegans*, germ cells remain connected to somatic cells to constitute the core of the gonad (rachis), and transcriptionally inactive oocytes are supported by mRNA and protein transported from the nurse cells through intercellular canals [153–157]. A similar phenomenon appears to exist in mammals but has evolved differently, due perhaps to the long dormancy of the oocyte and two-step nature of the process. Direct and open-ended intercellular connectivity is evolutionary conserved during formation of germ-cell cysts before the formation of individual primordial follicles [158], whereas *corona radiata* cells seem to contribute in a nurse cell–like manner through transzonal projections. Given that the ultimate function of the ovarian follicle is to produce and release a single highly competent egg, the devotion of cumulus cells to protecting and nurturing the oocyte seems to be a logical evolutionary development.

## Conclusion

**Fig. 4:**
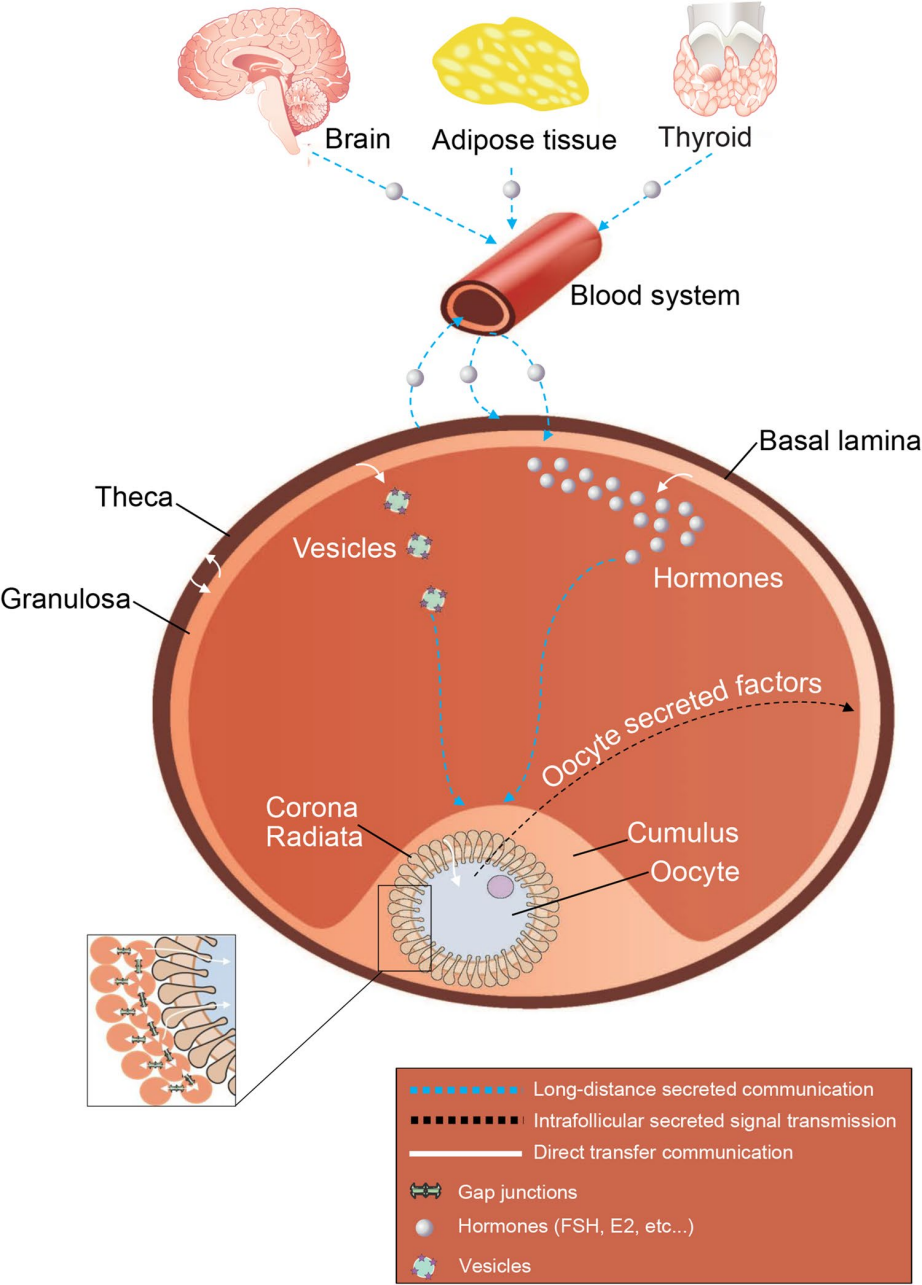
Schematic representation of the long-distance (wireless) and direct communications involved in managing folliculogenesis and oogenesis in mammals. Organs such as brain or thyroid send hormones to the follicle via the bloodstream. By binding to the somatic cell receptor of the follicle, the hormone enables its activation and the stimulation of its dependant signaling pathways. This endocrine signaling on the functions and the development of the follicle cells and the oocyte, without direct transmission by physical contact, constitutes a long-distance systemic (wireless) inward signaling route (represented by blue-dotted line). Moreover, by secreting extracellular vesicles containing miRNA, proteins and RNA, components involved in various pathway which are closely related to follicular growth and maturation, granulosa cells are another instance of secreted signaling into the local environment. The oocyte also actively communicates with its surrounding cells, using secreted factors (represented by dark dotted line), which therefore actively regulate the functions of the granulosa cells and the cumulus cells, related to growth and differentiation of somatic cells. Although much of the dialogue required for folliculogenesis and oogenesis occurs through secreted (wireless) communications, some inputs go through direct communications (represented by white arrows). This direct communication implies a direct transmission via physical contact. These direct material transfers occur between granulosa cells using gap junctions and between the oocyte and its surrounding cells using TZPs and gap junctions

It is clear that the biological success of the ovarian follicle relies on correct responses to signals between different groups of cells and from distant organs and on proper balance of cell growth and cell differentiation. Ovulation is the result of two interdependent systemic physiological events: folliculogenesis and oogenesis. It appears that paracrine communications intended for the oocyte and coming from the oocyte allow folliculogenesis and oogenesis to synchronize. The inward signals reach only cumulus cells, where direct cell-to-cell communication with the oocyte takes over. This transition needs further study (Fig. 4). It may be a necessity due to the inadequacy of long-distance systemic communication in certain cases since information is disseminated in a multicellular environment and messengers such as extracellular vesicles and miRNA have long half-lives. In addition, responding to the net sum of multiple messages may be less efficient than instant ON/OFF activation or stoppage of direct transfers of materials.

This direct control of the cumulus cells over the oocyte as observed by the control over meiosis resumption may suggest some level of physiological outsourcing of cellular fate. This may be necessary as the full-sized oocyte falls into transcriptional silence [159, 160]. In presence of an inactive nucleus, the oocyte becomes less responsive to incoming messages. Responses at this point could be based on post-transcriptional events such translation of stored mRNA or activation of proteins through post-translational modifications. Another option would be to impart responsiveness to surrounding cumulus cells, which could then relay external signals to the oocyte in direct form through transzonal projections.
